# How macrophages use extracellular calreticulin to chase their prey

**DOI:** 10.1080/2162402X.2025.2533494

**Published:** 2025-07-12

**Authors:** Liwei Zhao, Peng Liu, Oliver Kepp, Guido Kroemer

**Affiliations:** a Centre de Recherche des Cordeliers, Université Paris Cité, Sorbonne Université, Inserm, Equipe labellisée par la Ligue contre le cancer, Institut Universitaire de France, Paris, France; b INSERM US23/CNRS UAR 3655, Université Paris-Saclay, Metabolomics and Cell Biology Platforms, Institut Gustave Roussy, Villejuif, France; c Department of Biology, Institut du Cancer Paris CARPEM, Hôpital Européen Georges Pompidou, AP-HP, Paris, France

**Keywords:** Asialoglycans, cancer, cell stress, immunotherapy

## Abstract

Recent findings reveal that macrophages actively control clearance by desialylating target cells via NEU1 and releasing cathepsin-cleaved calreticulin (CALR) to mark them for phagocytosis. This uncovers a dual role for CALR as immune activator or repressor, depending on its form and context, with distinct implications for cancer immunity.

Calreticulin (CALR) is a key chaperone localized in the endoplasmic reticulum (ER), playing essential roles in calcium homeostasis and protein folding.^1,2^ Beyond these canonical functions within the lumen of the ER, cell surface-exposed CALR serves as an evolutionarily conserved recognition signal, facilitating gamete fusion in both yeast and mammals.^3,4^ Moreover, surface CALR functions as an “eat-me” signal, enabling phagocytes to identify and engulf target cells. During homeostatic clearance, macrophages can detect this signal to silently remove CALR-tagged apoptotic cells, whereas in the course of immunogenic cell death (ICD), ER-stress-activated PERK promotes ERp57-assisted CALR surface exposure dictates antigen uptake by dendritic cells (DC), ultimately triggering T cell-mediated adaptive immune responses^5,6^ (Figure 1a). Of note, CD47 expressed on tumors can deliver a ‘don’t-eat-me’ signal that inhibits phagocytosis, allowing malignant cells to evade immune clearance. Moreover, not all forms of CALR support immune activation. Mutations such as CALR del52 and CALR ins5 in myeloproliferative neoplasms, as well as truncations like CALR E405* in solid tumors, that disrupt the C-terminal Lys-Asp-Glu-Leu (KDEL) ER-retention motif, leading to aberrant secretion via Golgi-mediated exocytosis can act as local immunological decoys, saturating receptors like CD91 on antigen-presenting cells.^7^ As a result, such mutant forms of CALR can impair DC phagocytosis, suppressing CD8^+^ T cell responses, thus undermining the efficacy of chemotherapy and immune checkpoint inhibitors (ICI). Conversely it has been shown that macrophages can expose CALR on their surface which acts as a critical signal for guiding the phagocytosis of cancer cells, even when the tumor cells themselves do not express CALR.^8^ Furthermore, macrophages have been shown to actively secrete CALR through cleavage of the KDEL motif which then binds to N-terminal asialoglycans exposed on the surface of target cells tightly regulated by the presence of sialic acid residues, thereby flagging them for phagocytosis.^9^

**Figure 1.**
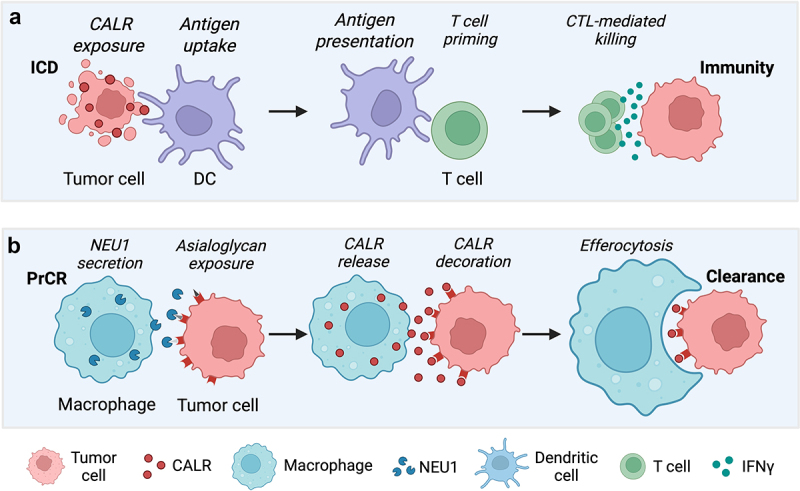
Multifaceted role of calreticulin (CALR) in immunity and clearance. (a) In the course of immunogenic cell death (ICD) endoplasmic reticulum (ER)-resident calreticulin (CALR) can translocate to the cell surface, where it functions as a conserved “eat-me” signal for antigen presenting dendritic cells (DCs) leading to adaptive immune responses. (b) Under homeostatic conditions surface exposed CALR promotes programmed cell removal (PrCR) by guiding macrophage-mediated phagocytic clearance of target cells. Recent findings further expand the role of macrophages in PrCR revealing that they actively orchestrate clearance by deploying neuraminidase (NEU1) to expose asialoglycans on target cells and releasing cleaved CALR, which binds these sites and marks cells for engulfment, underscoring macrophages as both orchestrators and executors of immune clearance.

A recent report from the group of Irving L. Weissman published in *PNAS* now reveals further insights into the molecular mechanisms of programmed cell removal (PrCR) positioning macrophages not only as executors but also as orchestrators of clearance.^10^ Here the authors described that macrophages were able to actively secrete the neuraminidase NEU1 to desialylate the cell surface of target cells while simultaneously releasing CALR through cathepsin-mediated cleavage of the KDEL motif. This two-step mechanism, which first unmasks asialoglycans and then overlays them with CALR generated a molecular “eat-me” signal that marked them for PrCR.^10^ This shift in perspective invites an illustrative metaphor. In the gladiatorial theaters of ancient Rome, the *retiarius* fought not with brute force but with finesse, using a net and trident to disarm and dispatch his opponent. The macrophage, in the biological arena, performs similarly. Its neuraminidase is the net, stripping protective sialic acids and exposing glycan vulnerabilities; cleaved CALR is the trident, striking the exposed site with precise, pro-phagocytic intent. The target is marked for phagocytosis and in turn cleared, not through violence, but via controlled unmasking and selective molecular tagging (Figure 1b).

Altogether, these findings underscore the dual and context-dependent role of CALR in immunity, acting either as a prophagocytic “eat-me” signal or as an immunosuppressive decoy depending on its cellular origin, its form and its localization. Clinically, this duality offers both a challenge and an opportunity: while surface-exposed CALR in cancer associates with favorable outcomes, high levels of soluble CALR may serve as a marker of therapeutic resistance. The identification of cathepsin B proteolytically facilitating CALR surface presentation, along with the deployment of the neuraminidase NEU1 by macrophages, now suggests that innate immune cells can actively shape the decision of which cells are cleared and which remain. However, until now the upstream signals governing this selectivity remained elusive. As Banuelos et al. highlighted, immune clearance is a highly orchestrated process requiring both precision and restraint. Harnessing this system for therapeutic benefit will require not only controlling the immunogenic functions of CALR but also avoiding its subversion by tumor intrinsic immunosuppressive signals. Therapeutically, combining agents that enhance CALR surface exposure with CD47 blockade on cancer cells may synergistically promote their selective clearance while minimizing off-target immune activation.

## Data Availability

Data sharing is not applicable to this article as no new data were created or analyzed in this study.
